# 60-nt DNA Direct Detection without Pretreatment by Surface-Enhanced Raman Scattering with Polycationic Modified Ag Microcrystal Derived from AgCl Cube

**DOI:** 10.3390/molecules26226790

**Published:** 2021-11-10

**Authors:** Jikai Mao, Lvtao Huang, Li Fan, Fang Chen, Jingan Lou, Xuliang Shan, Dongdong Yu, Jianguang Zhou

**Affiliations:** 1 Research Center for Analytical Instrumentation, State Key Laboratory of Industrial Control Technology, Institute of Cyber-Systems and Control, Zhejiang University, Hangzhou 310027, China; maojikai@zju.edu.cn (J.M.); 21937018@zju.edu.cn (L.H.); 11732041@zju.edu.cn (L.F.); 2 Department of Chemistry, Zhejiang University, Hangzhou 310027, China; kenobi@zju.edu.cn; 3 The Children’s Hospital Zhejiang University School of Medicine, Hangzhou 310000, China; jingan@zju.edu.cn; 4 Hangzhou Green Environment Science & Technology Co., Ltd., Hangzhou 310000, China; sxl@grean.com.cn; 5 Hospital of Zhejiang University, Hangzhou 310027, China; ddyu@zju.edu.cn

**Keywords:** 60-nt DNA direct detection, surface-enhance Raman scattering, Ag microcrystal, polycationic

## Abstract

Direct detection of long-strand DNA by surface-enhanced Raman scattering (SERS) is a valuable method for diagnosis of hereditary diseases, but it is currently limited to less than 25-nt DNA strand in pure water, which makes this approach unsuitable for many real-life applications. Here, we report a 60-nt DNA label-free detection strategy without pretreatment by SERS with polyquaternium-modified Ag microcrystals derived from an AgCl cube. Through the reduction-induced decomposition, the size of the about 3 × 3 × 3 μm^3^ AgCl cube is reduced to Ag, and the surface is distributed with the uniform size of 63 nm silver nanoparticles, providing a large area of a robust and highly electromagnetic enhancement region. The modified polycationic molecule enhances the non-specific electrostatic interaction with the phosphate group, thereby anchoring DNA strands firmly to the SERS enhanced region intactly. As a result, the single-base recognition ability of this strategy reaches 60-nt and is successfully applied to detect thalassemia-related mutation genes.

## 1. Introduction

Genomic DNA, located in the nucleus of our cells, is the basis of our genetic identity, controlling cellular functions, and the variations of DNA with epigenetic functions, which can lead to abnormal behavior of the cells, such as inherited hemoglobin disorders [1]. Therefore, the development of new methods to detect, profile, and sequence the alteration of the bases in genome and transcriptome has attracted much attention because of its potential to promote the development of gene diagnosis and treatment technology. Traditional DNA analysis methods, such as ^32^P-postlabelling [2], immunological detection [3,4], comet assay [5], and liquid chromatography-mass spectrometry [6] has obtained satisfactory experimental results, but the requirement of complex pre-processing such as fluorescent labeling, PCR amplification, or protonation, would cost considerable time and money.

As an alternative method, Surface-enhanced Raman has arisen as a powerful detection tool by its high sensitivity [7] and specific fingerprint information about a wide range of target molecules [8,9], especially since water has no Raman signal, SERS is widely used in the detection and structural characterization of biomolecular in a natural environment [10,11,12,13,14,15,16]. Thus far, the SERS readings of the vast majority of SERS-based DNA detection methods consist of extrinsic signals from Raman reporters or molecular sensors that undergo measurable alterations of their intensity and/or spectral distribution as a result of recognition events involving the specific targets [17], but it completely disregards the rich structure and chemical information of the SERS spectral fingerprint of the target DNA. Contrary to the expensive chemicals and complex chemistry required by the indirect method, the direct approach is the most straightforward layout, which can directly detect the distinctive SERS signal of the DNA strand absorbed on the surface of the nanostructured [18,19].

Direct mixing of DNA with plasma substrate (mainly Ag) does not produce a sufficiently strong or reproducible signal. According to the electromagnetic enhancement (EM) mechanism which is the dominant mechanism in SERS enhancement [18,20], the problem can be expressed as the synthesis of optimal and uniform size and is a highly aggregated SERS substrate, which could produce a wide area with stable and high EM field that allows DNA molecules to be firmly anchored on the surface. Until now, both kinds of silver nanoparticles (AgNPs) with opposite charges have been used to detect single-stranded [3,21,22] and double-stranded DNA [23,24,25]. In particular, the positively charged AgNPs adsorbed DNA by non-specific electrostatic interaction of the phosphate groups in the DNA backbone rather than by base-specific nucleoside–metal binding, the signal generated by it is more stable and sensitive, and reflects the true structural conformation of DNA [26,27,28,29]. However, for direct DNA detection, there are currently three challenges: First, whether the silver colloidal with positive or negative charge, the aggregate agents are necessary for cluster formation [30], but it is difficult to control the assembly of nanospheres into well-defined nanoparticle clusters with precise coordination numbers, severe batch-to-batch variation, and long aging-time limit-related applications. Second, direct SERS analysis of biomacromolecules, such as DNA, makes imperative the exposure of the target analytes to a large range of high EM fields in order not to restrict the detection to a small portion of the broad molecular structure [20]. Finally, in order to obtain high sensitivity and high repeatability in the SERS signal, it is necessary to firmly anchor the DNA on the metal surface with no selective adsorption for four bases. The adsorption capacity of small cations molecules such as spermine is still limited [19], which cannot detect high concentrations or long-stranded DNA.

**Figure 1:**
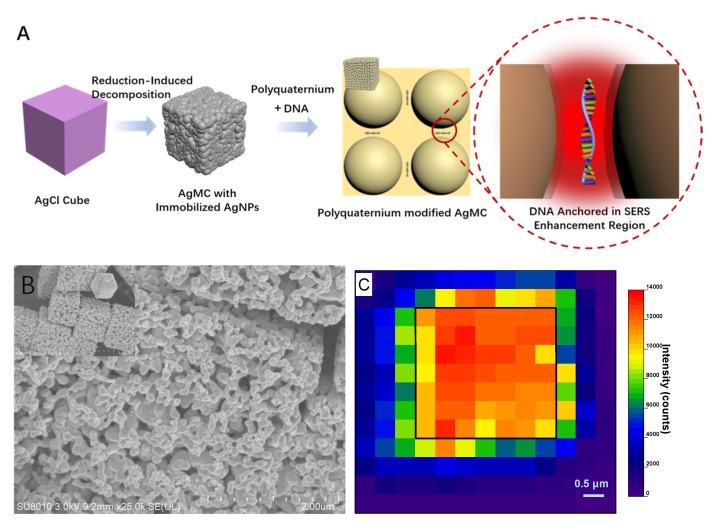
(**A**) Scheme of the synthesis and detection process of the poly@AgMC; (**B**) SEM image of the immobilized AgNPs stack on each other and dispersed on the surface; (**C**) SERS intensity mapping over an effective area of single AgMC (3 × 3 μm^2^) at a Raman signal of 734 cm^−1^.

Herein, to solve the difficulties mentioned above, we propose a 60-nt DNA SERS detection protocol without pretreatment that serves polyquaternium-modified Ag microcrystals (poly@AgMC) as SERS substrate, as illustrated in Figure 1A. The uniform size and shape of the AgCl cube will serve as the sole source of Ag and will be electrochemically reduced via a half-reaction of AgCl + e^−^→Ag + Cl^−^ to selectively dissolve Cl^-^, while the silver cation will turn into silver atoms, forming immobilized AgNPs on the surface. This type of reaction, termed reduction-induced decomposition (RID) [31], compared with the silver colloid obtained by reducing dissolved silver, has four advantages when it is used to directly detect DNA. First—a benefit from the surface-diffusion-controlled morphology evolution and simple stoichiometry of the compound—AgNPs have a more uniform and repeatable length scale; thereby, SERS signals generated by it are highly reproducible. Second, the size of AgNPs is controllable, and the optimal size for the highest EM can be obtained by adjusting the concentration of the reducing agent. Third, the obtained AgNPs are immobilized on the surface of the cube and are naturally kept in an aggregated state, which means that the most trivial aging step, the addition of aggregation agents, can be avoided, and the aggregation degree of the AgNPs will not be affected by ions in the complex environment. Finally, as a derivative of stability, the surface of the AgNPs are modified with polycationic molecules to enhance the non-specific electrostatic interaction with the phosphate group of DNA and yield the SERS spectra that represent the true composition of the DNA by anchoring the entire DNA in the vicinity of the nanostructures. Therefore, under the synergy between the AgMC and the polycationic, we are allowed to detect each nucleoside composition in the DNA stand by means of SERS. Moreover, its unique excellent ability in single-base recognition and anti-interference has promising application potential that can be applied in the detection of different hereditary diseases. In this work, we demonstrated the application of this SERS substrate to the detection of thalassemia-related DNA mutation.

## 2. Results

### 2.1. Synthesis of the Ag Cube with Immobilized AgNPs

Affected by the surface potential energy, the branches of the outward-growing crystal are easier to reduce; thus, the uniform size of AgCl is a necessary prerequisite for the synthesis of the uniform size of AgNPs. In this assay, tetrapropylammonium chloride (TPAC) not only serves as the sole source of Cl^−^, but also serves as a morphology-controlled stabilizer for the synthesis of AgCl. By adjusting the concentration, as shown in Figure S1 in the Supplementary Materials, we obtain the uniform cubic AgCl with a size of about 3 × 3 × 3 μm^3^. Various concentrations of NaBH_4_ are used to reduce AgCl cubes, Figure S2 in the Supplementary Materials. And we tested Raman intensity of AgCl cubes reduced by different concentrations of NaBH_4_ in the detection of 5 × 10^−5^ M SS1, Figure S4A in the Supplementary Materials, the sequence of which is listed in Table 1, to obtain an optimal plasmonic substrate. The EDS elemental mapping images of reduced product in Figure S3 in the Supplementary Materials suggest that AgCl cubes have been transformed into Ag cubes. As the SEM image shows in Figure 1B, the AgNPs are stacked on each other and are dispersed evenly. The AgNPs aggregate naturally without additional aggregating agents, thereby eliminating the most trivial step of the so-called “aging method”. Benefitting from the surface-diffusion-controlled morphology evolution and simple stoichiometry of the compound, the AgNPs formed by the rapid RID possess a uniform, reproducible length scale, independent of the precursors. The size of the nanoparticles, Figure S4B in the Supplementary Materials shows that the obtained AgNPs are normally distributed in the 63 nm range with a standard deviation (σ) of 9 nm, and can generate the optimal SERS enhancement. Furthermore, the molecular conformation, orientation, and binding specificity are important causes of SERS intensity fluctuations, especially for biological macromolecules, such as DNA, with a relatively large molecular mass. Given that the AgNPs are fixed, we modified a layer of polycationic polyquaternium molecules on its surface, which can firmly adsorb longer strands of DNA that are intact in the EM area. To characterize the successful modification of the polyquaternium, we measured the proportion of its unique sulfur element, which increased from 0.000% to 0.193% by EDS, Table S1 in the Supplementary Materials. In addition, the performance of polyquaternium have also been confirmed by comparing the Raman intensity of the AgMC modified with different charges in the detection of 5 × 10^−5^ M SS1, Figure S5 in the Supplementary Materials.

### 2.2. Performance as SERS Substrate

Repeatability and sensitivity are the two most important indicators in SERS detection. To confirm the SERS spectra of DNA obtained by this plasmonic substrate, which are highly robust, a 6.5 × 6.5 μm^2^ area was mapped by point-to-point scanning, with a step size of 0.5 μm during laser excitation. The signal intensity at 734 cm^–1^ of 5 × 10^−5^ M DNA was measured, excluding the intensity change caused by the incomplete coverage of the light spot when scanning the edge of the substrate. As shown in Figure 1C, the average peak intensity generated by the laser irradiation on the cube surface is 12,181.6 counts, with a relative standard deviation (RSD) of only 6.92%. In addition, we selected the center position of the surface of 20 cubes for sampling, and the obtained SERS spectrum verified our previous statement, which has a high degree of repeatability, Figure S6 in the Supplementary Materials.

### 2.3. Performance in DNA Detection

**Figure 2:**
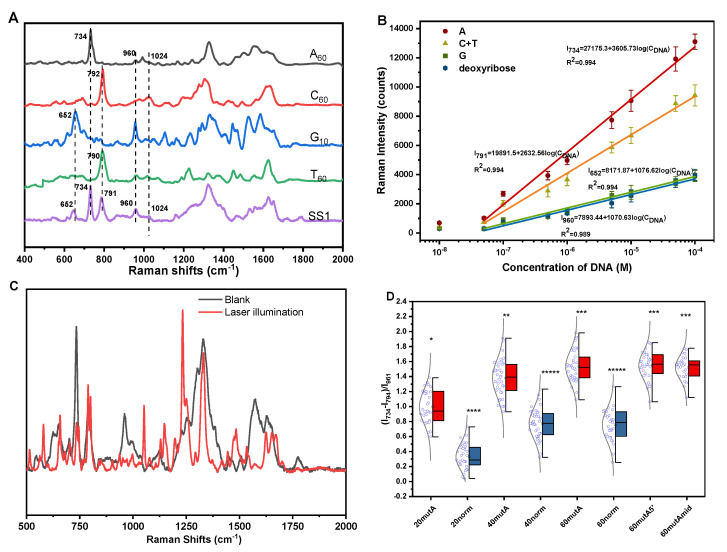
(**A**) Normalized SERS spectrum of homopolymeric DNA sequence and SS1. The characteristic peaks of each base of SS1 have been marked by comparison by the spectrum of homopolymeric DNA. (**B**) Linear relationship between the concentration of 60–nt DNA (SS1) and the Raman intensity of each base. Peaks at 652, 734, 791, 960 cm^–1^ are assigned to guanine (G), adenine (A), thymine (T), cytosine (C), and deoxyribose (dR), respectively. (**C**) SERS spectra change of the DNA adsorbed on the AgMC surface after irradiation by 633 nm laser. (**D**) Statistical analysis and distribution of the ratio after C mutated to A in different lengths of DNA sequence and position.

Definitive peak assignment is necessary for direct detection of DNA. Here, we selected four kinds of 60-nt homopolymeric DNA sequences for SERS experiments to assign the characteristic peaks in the Raman spectra of four kinds of nucleotide. The normalized SERS spectra of four kinds of DNA sequences and SS1 are shown in Figure 2A. Vibrational assignment of DNA is based on the literature reference and comparative spectra analysis. We selected the characteristic peaks of each base to illustrate. There are many common peaks from 900 to 1100 cm^–1^, which are sensitive to the deoxyribose–phosphate chain backbone structure and helix conformation. In this assay, the peak of 960 cm^−1^ assigned to the vibration of dR was the most obvious and stable appearance in the SERS spectrum. In the spectra of A60, the peaks at 1150–1700 cm^–1^ overlapped, which was attributed to the in-plane vibrations, such that the peak of 734 cm^–1^ was selected as the characteristic peak of adenine, which was assigned to the ring skeleton vibration. Guanine was featured by the peak at 652 cm^–1^, which was assigned to the ring breadth. In the spectra of C60, there was a well-separated peak at 792 cm^−1^, but when thymine appeared, the 790 cm^–1^ peak of thymine overlapped with it. The assignment of other peaks in the spectra is illustrated in detail in Table S2 in the Supplementary Materials.

High sensitivity is a major feature of SERS, according to the peak assignment as described above> Here, we studied the linear range in DNA detection using this SERS substrate. As shown in Figure 2B, we plotted the correlation curve of the characteristic peak intensities of each base and deoxyribose (dR) as the concentration of 60–nt DNA. When there is a high concentration of DNA (10^−4^ M) in the solution, due to the stronger electrostatic effect, the domination by the Stokes mode of adenine does not occur, which is caused by the orientation of DNA extended from the metal surface [3,4]. However, taking the strongest peak of 734 cm^–1^ as a reference, the benefit from the synergic effect of the AgMC and polycationic molecules, the detection limit of 60-nt DNA reaches 6.7 × 10^−8^ M (3σ/S). Since we only needed 10 μL of DNA solution to complete detection, in actuality, the SERS signal coming from 1.24 × 10^3^ DNA molecules was successfully detected, Section S4 in the Supplementary Materials. Further evidence comes from the observation of a sudden spectra change (Figure 2C). Photochemical decomposition or photobleaching was significantly reduced due to extremely few molecules adsorbed on the metal surface. After the DNA molecules adsorbed on the surface of AgMC were irradiated by the laser (λ = 633 nm) for 3 min, the spectra changed considerably, e.g., the peaks at 734 and 790 cm^–1^ happened to split into two. Since adenine adsorbed more firmly on the metal surface than the other bases, the peak intensity of adenine decreased considerably as well. At the same time, due to the strong effect of photochemical reduction, the peak of deoxyribose disappeared and the peak intensity of the phosphate group (1050 cm^–1^) became strong.

Although we have introduced high reproducibility and ultrasensitivity of poly@AgMC, the orientation of DNA bases adsorbed on the metal surface was difficult to constrain. Therefore, it was necessary to use a common peak existing in the four nucleotides as an internal standard to avoid the fluctuation of the SERS peak in quantitative analyses. Compared with the peak of 1024 cm^–1^ assigned to the symmetric stretching vibration of phosphodioxy, dR was closer to the bases; therefore, in the quantitative analysis, it is more desirable to take the peak of 960 cm^–1^ as the internal standard to avoid fluctuation of the SERS intensity. As long as one base is replaced, for example, cytosine replaced by adenine, the ratio is stable and normally distributed, and there is a significant difference between the DNA before and after the replacement (Figure 2D). Then, we extended the length of the DNA strand to assess the substrate’s capacity to recognize single bases. Due to the high reproducibility, high sensitivity, and strong electrostatic adsorption of the SERS substrate to avoid the orientation of DNA extending on the metal surface, we could better distinguish the Raman signal of different bases in longer DNA strands, and the ability of single-base recognition reached 60 bases. The ratio of each base was constant. By extending the length of the DNA strand, the ratio value of (I_734_ − I_791_)/I_960_ happened to increase. The reason we hypothesized is that the coordination bond between adenine and the metal is stronger than other bases under the action of base stacking power while other bases are far away from the metal surface; thereby, the intensity decreased. Finally, to avoid the possible impact of the substitution position on the result, we replaced the 5′-end and the middle of the DNA strand. The experimental results are consistent with the 3′-end replaced results.

When the solution is rich in various ions, it is easy to cause aggregation in the Ag colloid or instability of the aggregate degree. However, ions such as Na^+^ and Mg^2+^ are often added into the solution to maintain the stability of the DNA structure. To exhibit the excellent anti-interference ability for DNA detection in a complex environment, we introduced 100 mM of different interfering ions, listed in Figure S7 in the Supplementary Materials. As we can see, there is no significant difference after adding interferents.

### 2.4. Beta-Thalassemia Mutation Detection

**Figure 3:**
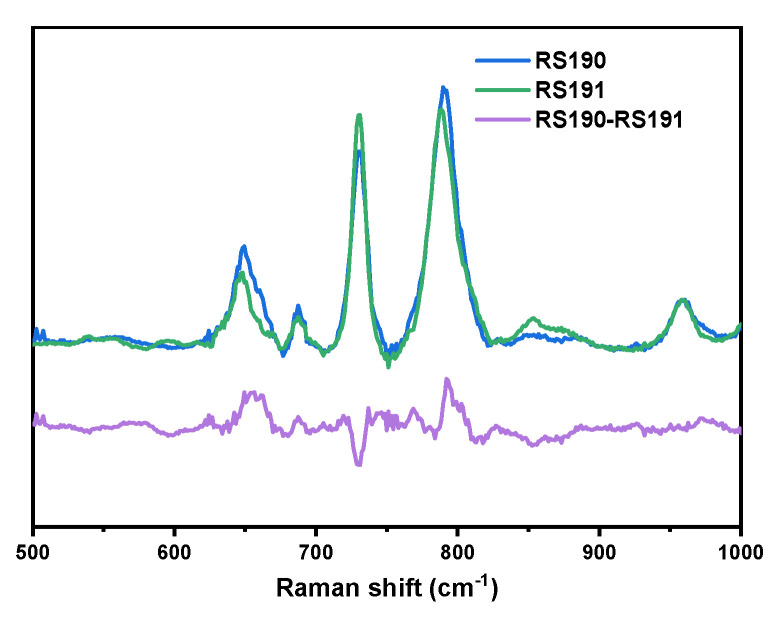
The normalized Raman spectrum and difference spectrum of the beta–thalassemia–related DNA sequence.

Although this method cannot distinguish the position of a specific base, it can effectively detect single-base mutations of genes. The specific-sequence oligonucleotide probe is a widely used technique for the detection of genetic mutations, and it has been proven that the optimal oligonucleotide length for best sensitivity and specificity ratios in transcriptome studies is 60–mers [2]. To reflect the potential of poly@AgMC in the detection of hereditary diseases, here, we chose to detect two kinds of gene mutations of the 60-nt sequence DNA related to the synthesis of beta-globin. The error transcription will cause globinogenesis anemiawe, which is also known as beta-thalassemia [32]. As shown in Figure 3, the spectrum of the two kinds of mutations was normalized by the peak intensity at 960 cm^–1^. The difference between RS190 and RS191 is that the former has one G and one C base more, but one A and one T base less; thus, the 734 cm^−1^ intensity of RS190 is lower than RS191, and the 652 cm^–1^ intensity is stronger. At the same time, although the characterization peak of C and T overlap, the Raman activity of C and T is different [3], which leads to the 791 cm^−1^ intensity of RS190 being stronger than RS191.

## 3. Materials and Methods

### 3.1. Materials

All reagents and solvents were purchased from commercial sources and used as received without further purification. Chemicals used in this study included tetrapropylammonlum chloride, ethylene glycol (EG, ≥99%), sliver nitrate (AgNO_3_, A.R. 99.8%, Sinopharm Group Chemical Reagent Co., Ltd., Shanghai, China), polyquaternium, and sodium borohydride (NaBH_4_, AR, 98%), which were purchased from Aladdin. TE buffer was purchased from Solarbio and stored at 4 °C until required. DNA oligonucleotides were purchased from Shanghai Sangon Biotechnology Co. Ltd. (Shanghai, China). The DNA sequences are listed in Table 1. DNA dry powder was stored at −20 °C. The stock solutions of each oligonucleotide were prepared in TE buffer and stored at 4 °C until use.

### 3.2. Synthesis

#### 3.2.1. Synthesis of AgCl Microcubes

For a typical synthesis of uniform AgCl microcubes, 1.4 mL of 2 M tetrapropylammonlum chloride aqueous solution and 20 mL ethylene glycol (EG) were added to a 50 mL flask. The mixture was stirred for 5 min in order to obtain a homogeneous solution. Subsequently, 180 uL of 1 M AgNO_3_ solution was added immediately into the mixture under stirring for 5 min. The mixture was placed in an oil bath at 190 °C with vigorous stirring for 30 min. After 30 min of reaction, heating was switched off and the mixture cooled to room temperature. Next, the supernatant fluid was collected and centrifuged at 16,000 rpm for 5 min, and the products were ultrasonically dispersed in ethanol and then washed. The prepared AgCl microcubes were dispersed in ethanol solution for further use.

#### 3.2.2. Reduction of Cubic AgCl

The samples prepared above were configured into 1 mg/10 μL solution. Taking 5 L of the above solution and mixing it with 1 mL of 7 mM NaBH_4_ solution, the reaction was vigorously stirred for 30 min. After 30 min, the resulting product was first centrifuged at 8000 r/min for 5 min to remove the supernatant and was then washed three times with water. The product was ultrasonically dispersed in water for further use.

#### 3.2.3. Modification of Polyquaternium

The synthesized silver cubes were mixed with 1 mL of 5 μL/10 mL polyquaternium at room temperature overnight. Then, the resulting product was centrifuged at 8000 rpm for 5 min to remove the supernatant and was washed three times with water. The sample was mixed with 20 uL water, sonicated for 1 min, and stored at room temperature for further use.

### 3.3. The SERS Detection

For SERS studies, 5 μL of TE buffer of DNA and 5 μL poly@AgMC were mixed and incubated at room temperature for 1 h before SERS measurements.

### 3.4. Equipment and Parameter Settings

The morphologies of the prepared AgCl microcubes were characterized by scanning electron microscope (SEM, 3.0 kV, SU-70, Tokyo, Japan). A confocal Raman spectrometer (Horiba HR Evolution 800, Tokyo, Japan) was used to record Raman spectra. The spectral resolution was less than 0.35 cm^−1^, and spectral repeatability was less than 0.03 cm^−1^. A 633 nm laser was focused onto the sample by a long working distance objective (100×, 0.90 NA); the incident laser power was 10 mW with a 2.5% NA filter. The acquisition time was 5 S, accumulated 3 times. The obtained data was analyzed using spectral analysis software package LabSpec 6.

## 4. Conclusions

In summary, we demonstrated the ability of poly@AgMC with ultra-sensitivity, high reproducibility, strong anti-inference, and single-base recognition of 60-nt DNA when used for direct DNA detection. Through the RID reaction, Ag cations are reduced to AgNPs, which are evenly immobilized on the surface of the cube. The average size is 63 nm, and the σ is 19 nm, which both meet normal distribution such that about 3 × 3 μm^2^ of the surface of AgMC becomes an ideal SERS substrate, with enough area to provide a strong and stable EM. Since the AgNPs were fixed, the polyquaternium was used to modify the surface to have more cations, enhancing the adsorption of the entire long DNA strand within the EM fields, achieving quantitative analysis of 60-nt DNA from 10^−4^ to 6.7 × 10^−8^ M. Not only that, the natural environment where DNA exists often contains high concentrations of ions, and various pre-processing of DNA such as PCR requires the addition of various ions. The poly@AgMC has a broad application prospect due to its strong anti-interference. Finally, we used the two mutation genes of beta-thalassemia as an example, successfully distinguishing the differences in the SERS spectrum, which exhibits great potential in DNA detection.

## Figures and Tables

**Table 1:** Oligonucleotide sequence used in the study.

Name	Sequence
A60	AAAAAAAAAA AAAAAAAAAA AAAAAAAAAA AAAAAAAAAA AAAAAAAAAA AAAAAAAAAA
C60	CCCCCCCCCC CCCCCCCCCC CCCCCCCCCC CCCCCCCCCC CCCCCCCCCC CCCCCCCCCC
G10	GGGGGGGGGG
T60	TTTTTTTTTT TTTTTTTTTT TTTTTTTTTT TTTTTTTTTT TTTTTTTTTT TTTTTTTTTT
SS1	AGATCAGGTCAGTTCAGCTCAGATCAGGTCAGTTCAGCTCAGATCAGGTCAGTTCAGCTC
20norm	AGATCAGGTCAGTTCAGCTC
20mutA	AGATCAGGTCAGTTCAG**A**TC
40norm	AGATCAGGTCAGTTCAGCTC AGATCAGGTCAGTTCAGCTC
40mutA	AGATCAGGTCAGTTCAGCTC AGATCAGGTCAGTTCAG**A**TC
60norm	AGATCAGGTCAGTTCAGCTC AGATCAGGTCAGTTCAGCTC AGATCAGGTCAGTTCAGCTC
60mutA	AGATCAGGTCAGTTCAGCTC AGATCAGGTCAGTTCAGCTC AGATCAGGTCAGTTCAG**A**TC
60mutA5′	AGATCAGGTCAGTTCAG**A**TC AGATCAGGTCAGTTCAGCTC AGATCAGGTCAGTTCAGCTC
60mutAmid	AGATCAGGTCAGTTCAGCTC AGATCAGGTCAGTTCAG**A**TC AGATCAGGTCAGTTCAGCTC
RS190	TGGGCAG**G**TTGG**C**ATCAAGCCCACAGGGCAGTAACGGCAGACTTCTCCTCAGGAGTCAG
RS191	TGGGCAG**A**TTGG**T**ATCAAGCCCACAGGGCAGTAACGGCAGACTTCTCCTCAGGAGTCAG

## Data Availability

Not applicable.

## Supplementary Materials

The following are available online: Figure S1: The SEM images of the morphology of the AgCl cubes, Figure S2: (A–E) The SERS images of different degrees of reduction. The AgCl cubes were reduced by 10^−1^ (A), 10^−2^ (B), 7 × 10^−3^ (C), 10^−3^ (D) and 5 × 10^−4^ M (E) concentration of NaBH_4_, respectively. AgNPs are the first to form fine particles on the surface (E), the adjacent AgNPs continue to aggregate to form larger particles (B, C, D), and finally form AgMC with a smooth surface (A), while increasing the concentration of the reducing agent NaBH_4_, Figure S3: The EDS spectrum of the unreduced AgCl cubes (A) and the AgMC was reduced by 7 × 10^−3^ M NaBH_4_ (B), Figure S4: (A) The Raman intensity of the AgCl cube was reduced by different concentrations of NaBH_4_ in the detection of 5 × 10^−5^ M of SS1. (B) Statistical analysis of the size of the AgNPs in a single AgMC, Figure S5: The Raman intensity of the AgMC were modified with different charges in the detection of 5 × 10^−5^ M SS1, Figure S6: The Raman spectrum of 20 Ag cube in the detection of 5 × 10^−5^ M SS1, Figure S7: Statistical analysis and distribution of SERS detection of different kinds of ions in DNA solution, Table S1: EDS statistical data of the AgCl cube and the AgMC reduced by 7 × 10^−3^ M NaBH_4_, Table S2: Peak assignment in DNA spectrum.
