# Supplementary material for: 60-nt DNA Direct Detection without Pretreatment by Surface-Enhanced Raman Scattering with Polycationic Modified Ag Microcrystal Derived from AgCl Cube
Source: Molecules. 2021 Nov 10;26(22):6790. doi: 10.3390/molecules26226790 (PMC8620099; doi:10.3390/molecules26226790)
Supplement: Supplementary file 1 [file molecules-26-06790-s001.zip › molecules-1396093-supplementary.pdf]

Supporting information

Section S1. Synthesis of AgMC with immobilized AgNPs on the surface

Section S1. Synthesis of AgCl cube

As the concentration of tetrapropylammonlum chloride increasing, the crystals continued to grow along the edges and corners, the branches of the crystal growing outward are easier to be reduced, causing the uneven SERS enhancement region on the surface. Here, we exhibit the uniformly cubic AgCl, the length of the cube is about 3 μm, after reduction, the size is sufficient to provide a SERS enhancement region for macromolecules.

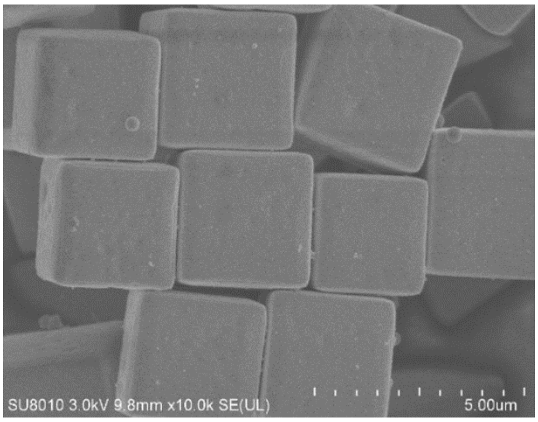

Figure S1. The SEM images of the morphology of the AgCl cubes.

Section S2. Synthesis of AgMC with immobilized AgNPs on the surface

After the reduction by the reducing agent $NaBH_4$, AgNPs gradually grow on the surface of the AgCl cube (Figure S2e), as described in the article, the reaction is so-called reduction-induced decomposition. As the concentration of $NaBH_4$ increasing, the AgNPs increased in size and number (Figure S2d) and continued to aggregate on the surface, highly curved ligaments well-connected to their neighbors (Figure S2b). With the concentration of $NaBH_4$ further increased, a shell formed on the surface (Figure S2a). Here, we demonstrated the ability to adjust the morphology of the AgNPs on the surface of the cube, just by adjusting the concentration of $NaBH_4$, we could obtain the optimal SERS substrate (Figure S2c).

Energy dispersive spectrometer (EDS) was used to study the elemental composition of the synthesized cubes, as shown in Figure S3 and Table S2, before reduction, the atomic ratio of Ag to Cl was 1.72, after reduction, the atomic percentage of Cl decreased to zero, in other words, the AgCl was completely reduced to Ag.

To confirm the performance on label-free DNA SERS detection, we mixed 5×10^{-5} M of SS1 with AgMC which was reduced by different concentration of $NaBH_4$, as shown in Figure S4, When the concentrations of $NaBH_4$ is too low, the size of AgNPs on the surface is too small; on the contrary, when the concentration of $NaBH_4$ is too high, there are too many AgNPs, interconnecting, forming large ligaments (Figure S4B) and even shells (Figure S4A). According to the theory of SERS electromagnetic enhancement, both of two aspects were bad for SERS enhance factor. When the concentration of NaBH4 reached 7 mM (Figure S2c), the size of AgNPs was 63 nm with the standard deviation σ is 9 nm, which is the optimal size for forming SERS enhancement, the intensity reached 4966.82 counts, the relative standard deviation was 10.87% (FigureS4a).

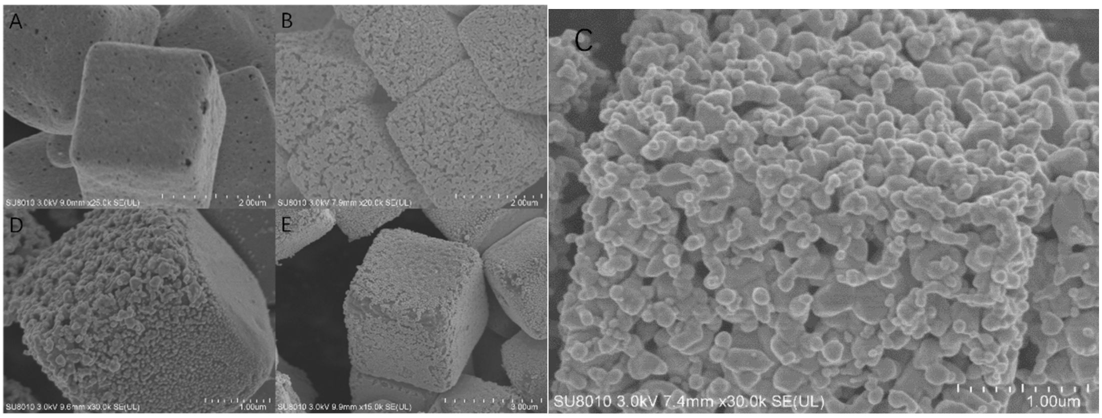

Figure S2. (A-E)The SERS images of different degrees of reduction. The AgCl cubes were reduced by 10^{-1} (A), 10^{-2} (B), 7×10^{-3} (C), 10^{-3} (D) and 5×10^{-4} M (E) concentration of $NaBH_4$, respectively. AgNPs are the first to form fine particles on the surface (E), the adjacent AgNPs continue to aggregate to form larger particles (B, C, D), and finally form AgMC with a smooth surface (A), while increasing the concentration of the reducing agent $NaBH_4$.

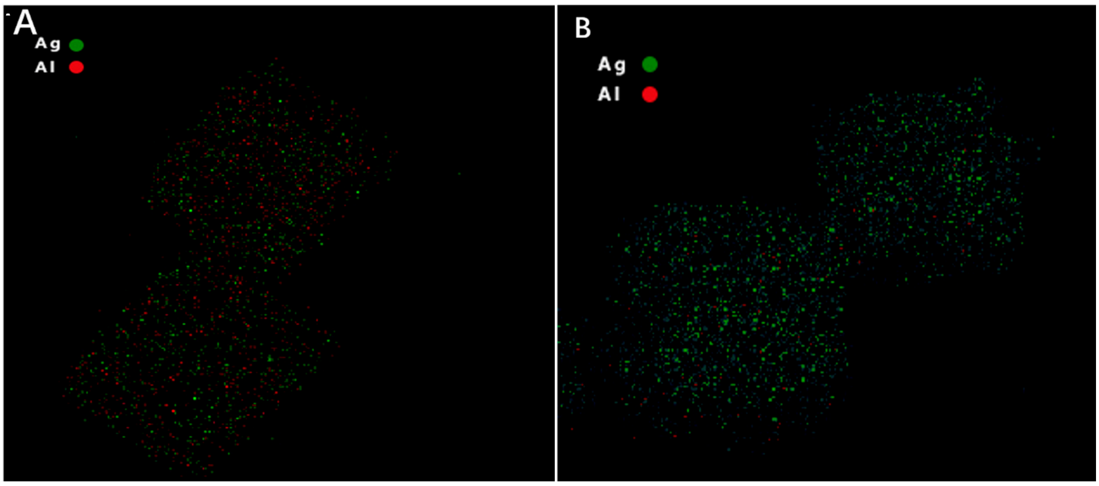

Figure S3. The EDS spectrum of the unreduced AgCl cubes (A) and the AgMC was reduced by 7×10^{-3} M $NaBH_4$ (B).

Table S1. EDS statistical data of the AgCl cube and the AgMC reduced by 7×10^{-3} M $NaBH_4$.

**Name**	**Elt.**	**Line**	**Intensity (c/s)**	**Atomic %**	**Atomic Ratio**	**Conc**	**Units**	**Error 2-sig**	**MDL 3-sig**
AgCl cube	S	Ka	0.00	0.00	0.000	0.000	wt.%	0.00	0.00
	Cl	Ka	76.74	36.71	1.000	16.01	wt.%	0.52	0.34
	Ag	La	89.50	63.29	1.724	83.99	wt.%	2.53	1.65
AgMC	S	Ka	0.42	0.193	1.000	0.057	wt.%	0.15	0.23
	Cl	Ka	0.00	0.000	0.000	0.000	wt.%	0.00	0.00
	Ag	La	145.5	99.81	516.8	99.94	wt.%	2.30	1.31

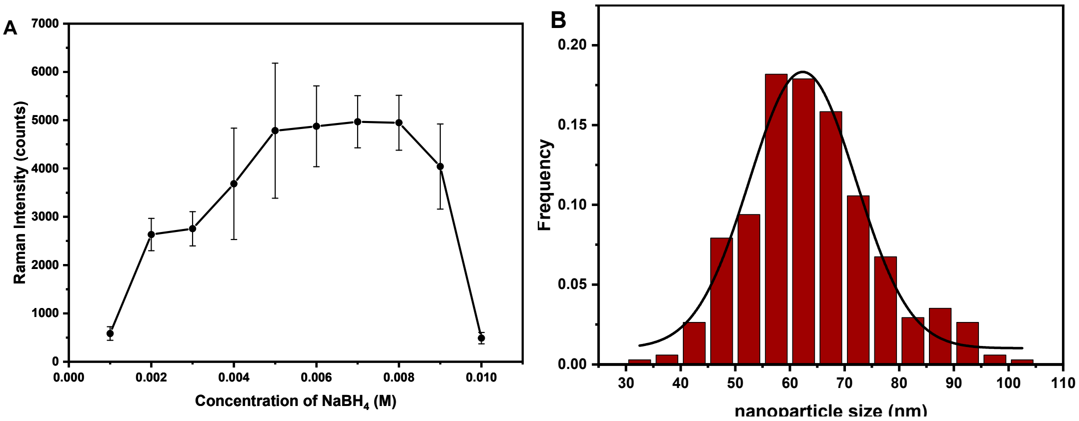

Figure S4. (A) The Raman intensity of the AgCl cube was reduced by different concentrations of $NaBH_4$ in the detection of 5×10^{-5} M of SS1. (B) Statistical analysis of the size of the AgNPs in a single AgMC.

Section S3. Modification of polycationic molecule

To prove that we successfully modified polycationic molecule polyquaternium on the surface of silver cube, EDS was used to measure the unique sulfur atom of polyquaternium. As shown in Table S2, compared to before, the percentage of atomic from 0.000 to 0.193%.

The role of the polycationic molecule was to provide a large number of cations and compared to the small molecule, producing a stronger non-specific electrostatic effect on the negative charged phosphate groups in DNA. Tetrapropylammonium chloride was used as the source of Cl^- and the morphology-controlled stabilizer for the synthesis of AgCl, to avoid additional variables, it was used to modify AgMC with positive charge as a representative of the small cationic molecule. Sodium dodecyl sulfate is a representative polyanionic molecule which used to modify the AgMC with negative charge. As shown in Figure S5, the adsorption effect of the polycationic molecule on DNA was obvious, take the peak at 734 cm-1 with the strongest intensity as a reference, compared to negative charge modified on the surface, the intensity increased more than 5 times, and compared to small cationic molecule, the intensity increased more than twice.

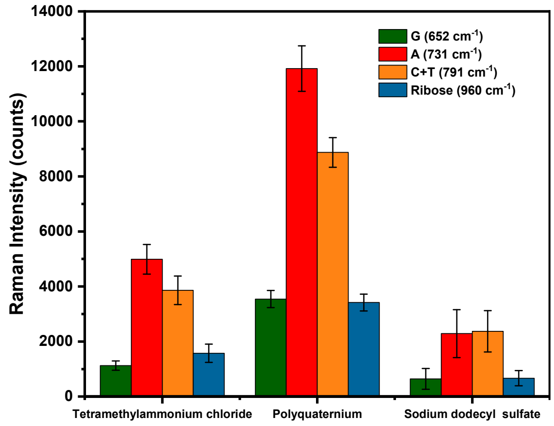

Figure S5. The Raman intensity of the AgMC were modified with different charges in the detection of 5×10^{-5} M SS1.

Section S4. Sensitivity of the SERS substrate

As described in the process of detection, without precise optimization, just 10 μL of DNA was

required to achieve detection, therefor, to reflect the high sensitivity of the substrate that closed to single-molecular detection, we did a calculate as follows:
For each detection process, the amount of DNA excited by laser is calculated by followed formula:

$$n_l = n_t \times \frac{m_s}{m_t} \times \frac{S_l}{S_t}$$

Where,

n_l is the molar mass of the DNA excited by laser;

n_t is the molar mass of all DNA;

m_s is the mass of one AgCl cube;

m_t is the total mass of AgCl in a measurement experiment.

S_l is the area of the laser spot;

S_t is the surface area of an AgMC;

The laser spot size is calculated by the following formula:

$$\mathbf{d} = \frac{4M^2\lambda f}{\pi D}$$

Where,

d is the light spot diameter;

M^2 is the beam mode parameter;

λ is wavelength;

f is lens focal length;

D is input beam diameter at the length.

The parameters corresponding to this instrument substituted into the formula:

$$\mathbf{d} = 1.22\lambda f$$

As a result, for 10 μL of 6.7×10^{-8} M DNA solution, even assuming that all DNA is adsorbed on the metal surface, the Raman signal was come from just 2.15×10^{-21} mol DNA, in other words, about 1.24×10^{3} DNA molecule could be successfully detected with AgMC as the SERS substrate. According to the literature(Nie and Emery 1997), only one out of perhaps 100 to 1000 particle is optically hot, therefore, we believed the enhancement effect of AgMC could achieved detection with high sensitivity that extremely close to single-molecule.

Section S5. Reproducible of the SERS substrate

The distribution of SERS enhancement at the different positions on the surface of a single AgMC has shown in the article (Figure 1c), here we show the SERS spectra of different AgMC, the number of samples was 20, take the peak at 734 cm^{-1} as a reference, the average peak intensity was 12771.0 counts, the relative standard deviation was only 7.84%.

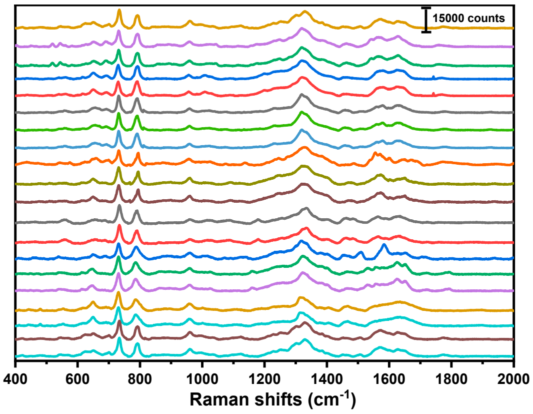

Figure S6. The Raman spectrum of 20 Ag cube in the detection of 5×10^{-5} M SS1.

Section S6. The ability of anti-inference

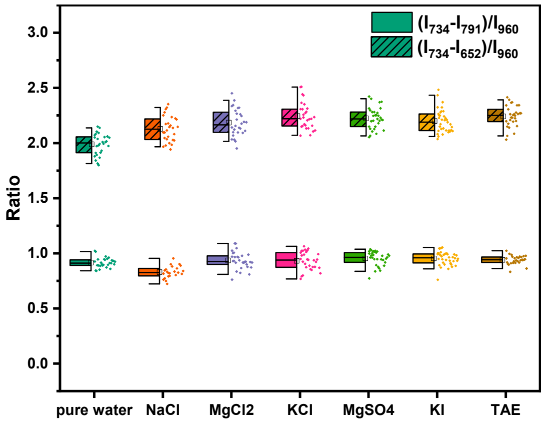

Name	$(I_{734}-I_{791})/I_{960}$	Standard Deviation	$(I_{734}-I_{652})/I_{960}$	Standard Deviation
Pure water	0.916	0.044	2.31	0.11
NaCl	0.830	0.054	2.47	0.13
MgCl2	0.935	0.077	2.53	0.14
KCl	0.931	0.082	2.60	0.14
MgSO4	0.954	0.065	2.58	0.11
KI	0.954	0.062	2.55	0.12
TAE	0.940	0.039	2.60	0.11

Figure S7. Statistical analysis and distribution of SERS detection of different kinds of ions in DNA solution.

Table S2 Peak assignment in DNA spectrum

Peak position (cm^{-1})	Assignment
541	A, wag C-H, N-H
613	Ring deformation
651	G, 5-ring deformation, 6-ring deformation, wagging NH_2
690	polyquaternium
734	A, ring breath
791	C/T, ring breath
961	Deoxyribose /PO_2^-
1024	Vibration of PO_2^-

1158	T/G
1247	G/T
1380	A/G
1395	G
1467	backbone
1530	A/G
1556	Base ring modes
1624	C/T
1649	G
1720	G，vibration of C=O

References:

Nie, S.M., Emery, S.R., 1997. Probing single molecules and single nanoparticles by surface-enhanced Raman scattering. SCIENCE 275(5303), 1102-1106.
